# Multiple lines of inhibitory feedback on AKT kinase in Schwann cells lacking TSC1/2 hint at distinct functions of mTORC1 and AKT in nerve development

**DOI:** 10.1080/19420889.2018.1433441

**Published:** 2018-02-12

**Authors:** Keit Men Wong, Bogdan Beirowski

**Affiliations:** a Hunter James Kelly Research Institute, Jacobs School of Medicine and Biomedical Sciences, University at Buffalo, Buffalo, NY, USA; b Department of Biochemistry, Jacobs School of Medicine and Biomedical Sciences, University at Buffalo, Buffalo, NY, USA

**Keywords:** myelin, mammalian target of rapamycin, tuberous sclerosis complex, PTEN, glia

## Abstract

During nerve development, Schwann cells (SCs) build multilayered myelin sheaths around axons to accelerate nerve conduction. The mechanistic target of rapamycin complex 1 (mTORC1) downstream of PI3K/AKT signaling lately emerged as a central anabolic regulator of myelination. Using mutant mice with sustained mTORC1 hyperactivity in developing SCs we recently uncovered that mTORC1 impedes developmental myelination by promoting proliferation of immature SCs while antagonizing SC differentiation. In contrast, mTORC1 stimulates myelin production, rather than SC proliferation, in already differentiated SCs. Importantly, these diametrical mTORC1 functions were unmasked under settings of greatly suppressed AKT signaling. Here we demonstrate, inter alia, additional mechanisms of feedback inhibition of AKT by mTORC1, such as strikingly elevated PTEN levels in SCs with disruption of the mTORC1 inhibitory complex, TSC1/2. These data lead us to propose a model wherein mTORC1 and AKT have distinct roles in developing SCs that have to be precisely coordinated for normal myelinogenesis.

The myelination of axons in the peripheral nervous system (PNS) relies on induction of excessive anabolism of Schwann cells (SCs) within a relatively short time window. During the first two weeks of postnatal development SCs produce massive amounts of compact myelin from expanding glial plasma membranes [1]. This anabolic challenge for SCs is largely triggered and then invigorated by molecular cues derived from axons and extracellular matrix components via receptor tyrosine kinases and G protein-coupled receptors [2] One of the prerequisites for myelination is preceding proliferation of SCs as long axons have to be populated by adequate numbers of glial cells. SC proliferation is also vital for normal radial sorting of axons, a process in which SCs extract larger axons from immature axon bundles to form a so-called 1:1 relationship between the SC and axon [3]. Yet, these processes have to be constrained to enable cell-cycle exit and properly timed differentiation of SCs. As the thickness of the axonal myelin sheath is optimized for fast nerve conduction, the subsequent growth of myelin sheaths also has to be strictly regimented. It remains only poorly understood which intracellular signal transduction pathways and control mechanisms regulate these aspects of SC development. Many hereditary and acquired neuropathies such as Charcot-Marie-Tooth disease and diabetic neuropathy due to nutrient overload are associated with abnormal SC proliferation, differentiation, and myelination. Thus, identifying the pathways and molecules involved would likely enhance therapeutic approaches toward nerve repair.

The phosphatidylinositol 3′ kinase (PI3K)/AKT and downstream mammalian target of rapamycin complex 1 (mTORC1) signaling pathway recently took center stage in numerous reports focusing on the molecular mechanisms of myelination (e.g. [4–23]). Collectively, these studies argue for a positive regulatory role of PI3K/AKT for myelin production, and this function is frequently attributed to the major downstream signaling output, mTORC1. mTORC1, a large multiprotein complex comprising the mTOR core kinase and several essential adaptor molecules, is known to increase multiple anabolic pathways including synthesis of proteins and lipids, which are the main constituents of myelin. In support, prominent hypermyelination was previously documented in mutant mice with SC-specific deletion of the phosphatase and tensin homolog (PTEN) that leads to increased AKT activity, as well as in mice with constitutively hyperactive AKT signaling in SCs [11–13]. This hypermyelination could be antagonized by pharmacological inhibition of the abnormally elevated mTORC1 activity in these mutants, implicating mTORC1 as the principal component of this signaling axis. However, the consequences of such aberrant mTORC1 signaling on early aspects of nerve development including proliferation and differentiation of SCs, and the onset of myelination, have not been studied until recently. Similarly, the impact of different levels of mTORC1 hyperactivity on these processes remained unknown.

We recently demonstrated that mTORC1 activity declines rapidly in mouse SCs while postnatal nerve development and myelination proceed [24]. This discovery dovetails with another recent study additionally demonstrating that this moderation of mTORC1 activity is paralleled by similarly decreasing AKT activity [4]. Together, these temporal patterns are at odds with the model that the PI3/AKT-mTORC1 axis promotes developmental myelination *in vivo,* which clearly occurs during the decline of signaling activity. We thus hypothesized that this pathway with mTORC1 as major signaling output may have a different function during SC development. To test this we sought to counteract the physiological downregulation of mTORC1 in developing SCs of mice by conditional disruption of the major upstream inhibitors of mTORC1, the tuberous sclerosis proteins TSC1 and TSC2, and the indirect inhibitor PTEN. This was accomplished by using a Cre line that functions in developing SCs (P0^Cre^ mice) [25]. TSC1 associates with TSC2 to form an active inhibitory signaling complex (TSC complex). Deletion of TSC2 or the less dominant component TSC1 eliminates the repressive effects of these proteins on mTORC1 via the small G protein Rheb [26]. On the other hand, PTEN inactivation results in TSC2 inhibition through multi-site phosphorylation of the TSC2 protein by increased AKT activity, and consequently mTORC1 induction [27]. As expected, mutant mice lacking TSC2 in developing SCs (TSC2^fl/fl^; P0^Cre^ mice ; TSC2-SCKO) displayed more pronounced mTORC1 hyperactivity as compared to mice with TSC1 (TSC1^fl/fl^; P0^Cre^ mice; TSC1-SCKO) or PTEN disruption (PTEN^fl/fl^; P0^Cre^ mice; PTEN-SCKO) [24]. Remarkably, only TSC2-SCKO mice developed a dramatic early-onset neuropathy due to the failure to myelinate peripheral nerve axons both in *in vitro* and in *vivo* paradigms of myelin development (Figs. 1, 2). Organotypic dorsal root ganglia (DRG) cultures from control or TSC2-SCKO mice prepared from E13.5 embryos showed markedly decreased TSC2 immunoreactivity in non-neuronal cells consisting mostly of SC precursors, while TSC2 levels in neurons were indistinguishable from control preparations (Fig. 1a). Such TSC2-deficient SC precursors were unable to myelinate DRG axons under myelination-promoting conditions including addition of ascorbic acid; this is demonstrated by a drastic reduction of MBP^+^ (myelin basic protein) neurite segments in comparison to control (Fig. 1b). Consistent with this *in vitro* model, serial analysis of peripheral nerves from TSC2-SCKO mice at different postnatal ages by semithin light- and electron microscopy revealed normal nerve structure directly after birth, but then a substantial deficiency of myelin formation resulting in a severe dysmyelination phenotype by the age of weaning (Fig. 2a, b). A progressive increase of SC nuclei per microscopic field was already evident in these mutant nerve preparations. Further evidence for elevated SC numbers and abnormal SC proliferation came from quantification of DAPI^+^ cells on frozen sciatic nerve cross sections (Fig. 3a), well in line with our earlier report [24]. As the nerve size remained indistinguishable from control samples (Fig. 3b), the aberrant SC numbers resulted in drastically increased cell densities in TSC2-SCKO nerves (Fig. 3c). Mechanistically, we demonstrated that the strongly sustained mTORC1 activity in TSC2-SCKO nerves rendered SC precursors unable to exit the cell cycle and differentiate into myelinating SCs [24]. This was accompanied by markedly decreased levels of the key cyclin dependent kinase (CDK) inhibitor p27^Kip1^ in TSC2-SCKO nerves, and our data suggest that elevated translation of cell cycling proteins which are known to suppress p27^Kip1^ levels account for this abnormality [24,28]. Alternatively, others postulated that mTORC1 hyperactivity and increased downstream signaling through the S6 kinase branch directly exerts inhibitory effects on the expression of the master transcriptional regulator of myelination Egr2/Krox20, and this largely explains the impaired SC differentiation and myelination [4]. However, it remains unclear in this model how the attenuated levels of Egr2/Krox20 lead to aberrant SC proliferation. Importantly, in our study, the milder mTORC1 hyperactivity in developing SCs in TSC1-SCKO and PTEN-SCKO mice was not sufficient to phenocopy the severe dysmyelination seen in TSC2-SCKO mice, arguing for dose-dependent effects of mTORC1 in the SC lineage. These mutant mice also displayed increased SC numbers and reduced myelin formation during early nerve development, albeit to a much lesser extent [24]. These data motivated us to propose that mTORC1 hyperactivity above a certain threshold in developing SCs is prohibitive for myelination [28]. If SC mTORC1 activity remains below this critic threshold, mutant SC precursors are principally capable of exiting the cell cycle and eventually mature into myelinating SCs as evidenced by the presence of abundant myelin profiles in TSC1-SCKO and PTEN-SCKO mutants [24]. In accord with this model, the treatment of TSC2-deficient mice with the allosteric mTORC1 inhibitor rapamycin rescued the abnormal SC proliferation and differentiation resulting in myelination recovery, which was accompanied by marked downregulation of the mTORC1 hyperactivity [24].

**Figure 1.**
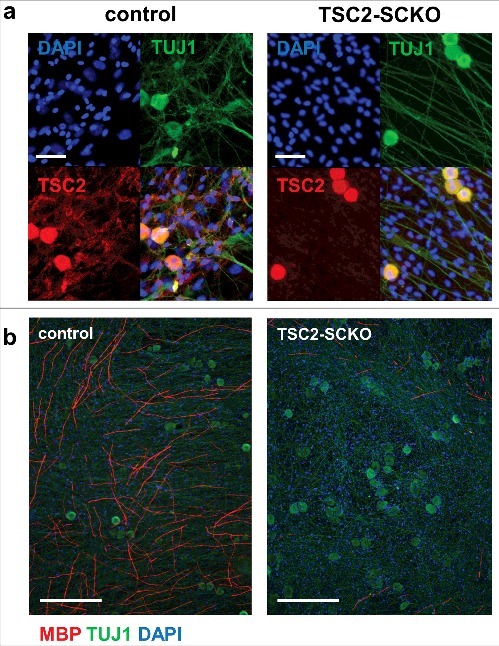
Arrest of myelination in DRG/SC co-cultures from TSC2-SCKO mice. A: Immunofluorescence of dorsal-root ganglion (DRG) neuron-SC co-cultures from control and TSC2-SCKO mouse embryos prior to induction of myelination (DIV 14) using the indicated antibodies. Note abundant TSC2 labeling of TUJ^+^ neuronal cell bodies and axons, and near absence of TSC2 signals in interstitial DAPI^+^ SC precursors prepared from TSC2-SCKO embryos. This indicates abolished TSC2 protein expression exclusively in mutant SCs. Scale bars: 50 µm. B: immunofluorescence of dorsal-root ganglion (DRG) neuron-SC co-cultures from control and TSC2-SCKO mouse embryos under myelination conditions (DIV 18, 11 days after ascorbic acid addition). Note drastic reduction in the number of MBP+ neurite segments in the mutant. Scale bars: 250 µm.

**Figure 2.**
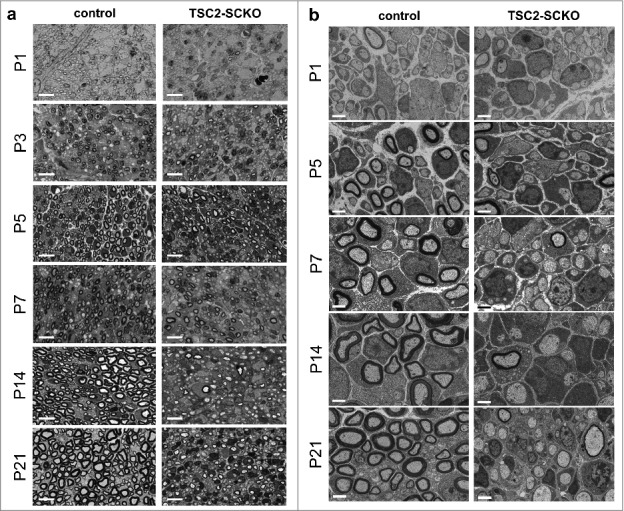
Arrest of myelination in nerves from TSC2-SCKO mice. Semithin light (A) and electron microscopy (B) of transverse sciatic nerve sections from control and TSC2-SCKO mice at the indicated ages showing a dramatic reduction in nascent myelinated fibers during development, so that most axons remain unmyelinated in the mutant. The reduced myelination is already prevalent at P3/P5. Scale bars: 10 µm (A), 2 µm (B).

**Figure 3.**
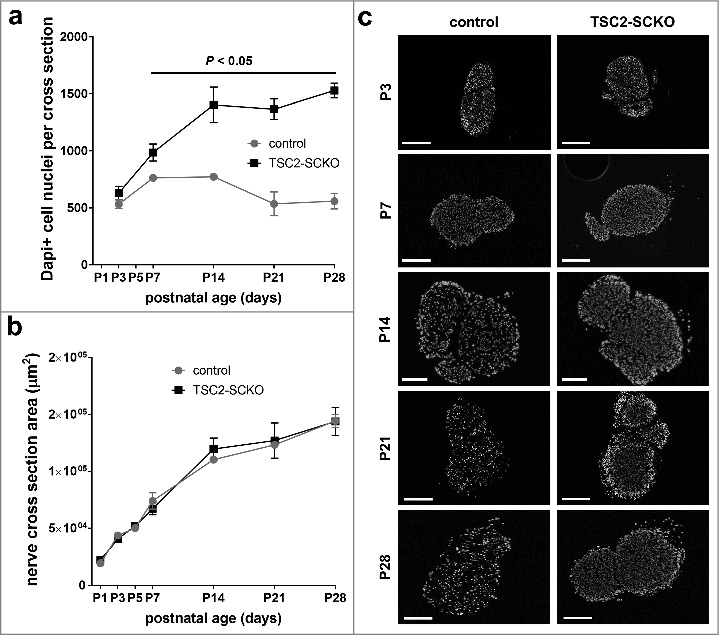
Hyperplasia in TSC2-SCKO sciatic nerves. A: Quantification of DAPI^+^ cell nuclei in sciatic nerve cross sections from control and TSC2-SCKO mice at the indicated postnatal ages showing progressive hyperplasia in the mutant. N = 3 mice per genotype at each age. B: Quantification of sciatic nerve cross section areas in control and TSC2-SCKO mice at the indicated postnatal ages demonstrating similar nerve sizes despite dysmyelination in the mutant. N = 3–4 mice per genotype at each age. C: Representative fluorescence microscopy of transverse sciatic nerves sections from control and TSC2-SCKO mouse at the indicated ages showing progressive elevation in the total numbers of DAPI^+^ cell nuclei as well as cell densities in the mutant. Scale bars: 250 µm (1-2^nd^, 4–5^th^ row), 100 µm (3^rd^ row).

Importantly, during the course of our studies we showed that nerves from TSC2-SCKO and TSC1-SCKO mice showed dramatically reduced AKT activity in SCs as demonstrated by the reduction of two widely used activity readouts (phosphorylation of AKT at Thr308 and Ser473) [24]. We ascribed this to a well-established inhibitory feedback loop that suppresses the scaffolding adaptor protein insulin receptor substrate 1 (IRS-1) by constitutively active S6 kinases downstream of mTORC1 [29]. Under physiological conditions IRS-1 promotes PI3K signaling leading to activation of PDK1 (Phosphoinositide dependent kinase 1) that then phosphorylates and activates AKT (phosphorylation at Thr308). In support of this feedback loop being activated, the protein levels of IRS-1 were drastically downregulated in TSC2-SCKO nerves (Fig. 4a). Additionally, the mTORC1 substrate Grb10 (growth factor receptor-bound protein 10) has been implicated in the suppression of IRS-1 expression [30]. and we found slightly increased protein levels of Grb10 in TSC2-SCKO nerves (Fig. 4b).

**Figure 4.**
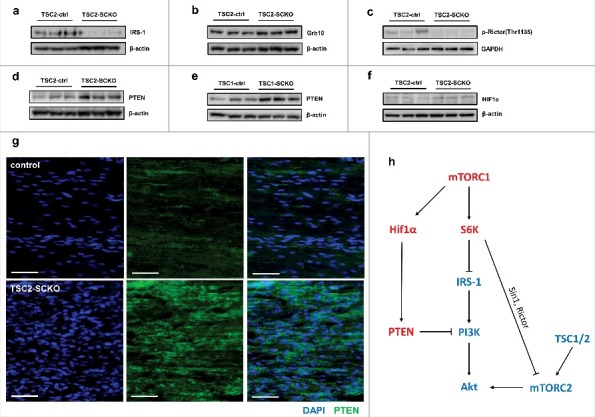
Multiple distinct mechanisms for downregulation of AKT activity in TSC1/2-deficient SCs. A-F: Western blots of sciatic nerve lysates from control and TSC2-SCKO/TSC1-SCKO mice at age P28 (A-E) or P14 (F) (3 mice per group), probed with the indicated antibodies. G: Immunofluorescence of longitudinal frozen sciatic nerve sections from control and TSC2-SCKO mice (age P28) showing greatly increased immunoreactivity of PTEN in the mutant (cytoplasmic signals surrounding DAPI+ nuclei). Scale bars: 50 µm H: Schematic summarizing distinct mechanisms underlying the downregulation of AKT activity in SCs from TSC1/2-SCKO nerves. Components highlighted in red are upregulated while blue highlighting depicts downregulation in mutant nerves. Note downregulation of insulin receptor substrate 1 (IRS-1) by constitutively active S6 kinases downstream of mTORC1. This leads to attenuated activation of PI3K/AKT. Lack of mTORC2 stimulation occurs through abolished TSC1/2 which results in reduced AKT phosphorylation in position Ser473 as well as through feedback inhibition by S6 kinases. Lastly, hyperactive mTORC1 promotes Hif1α transcription factor expression which results in increased *PTEN* transcription, and thus attenuated PI3K/AKT signaling.

Moreover, it has been shown that TSC1/2 interacts with mTORC2, the other key mTOR kinase complex besides mTORC1, and supports the activity of the mTOR core kinase in this multiprotein aggregate [31,32]. The mTORC2 complex is known to be the kinase of AKT at the serine in position 473, thus stimulating AKT activity. Hence, disruption of TSC1 or TSC2 results in diminished mTORC2 activity and therefore contributes to reduced phosphorylation of AKT. Alternatively, inhibition of mTORC2/AKT activity may occur through S6K1-mediatied phosphorylation of Sin1 (i.e. mTORC1-induced feedback), an essential component of the mTORC2 complex [33]. Indeed, it has been shown that TSC2 depletion in mouse embryonic fibroblasts (MEFs) results in increased phosphorylation of Sin1 at two sites (Thr86 and Thr398), a modification that dissociates Sin1 from the mTORC2 complex and impairs its integrity [33]. This is in keeping with earlier studies having demonstrated that Sin1 is indispensable for AKT activation by the mTORC2 complex [34].

Reasoning along the same lines, we then assessed site-specific phosphorylation at Thr1135 of the mTORC2 component Rictor that has been suggested as another feedback pathway downregulating mTORC2 and thus AKT activity [35–37]. Additionally, this Rictor phosphorylation site has been used as an mTORC2 activity marker [38]. We found reduced phosphorylation of Rictor at Thr1135 in TSC2-SCKO nerves (Fig. 4c), a site that has been proposed to be directly phosphorylated by mTORC1-dependent kinase S6K1 [35,37,39]. This is a surprising observation because S6K1 is hyperactivated in TSC2-deficient SCs [24]. However, other AGC family kinases including AKT1 and SGK1 may phosphorylate above Rictor site as well, [35,40]. and these activities are likely attenuated in TSC2-deficient SCs. On the other hand, some authors report no or only minor effects on mTORC2 activity by Thr1135 phosphorylation of Rictor [37,41].

Finally, because PTEN is the most potent negative regulator of PI3K/AKT signaling, we studied its protein levels in TSC2-SCKO and TSC1-SCKO nerves. Unexpectedly, we found that these mutant nerves show substantially elevated levels of PTEN (Fig. 4d, e, g). Precedents for PTEN increases in TSC2-deficient cells have been reported, and it has been proposed that the TSC complex inhibits expression of PTEN through a canonical hypoxia-responsive element on the *PTEN* promoter that can be occupied by Hif1α [42,43]. In support of such function, TSC2-SCKO nerves showed increased protein levels of the Hif1α transcription factor (Fig. 4f), the expression of which is known to be positively regulated by mTORC1. Hence, the increased Hif1α abundance likely promotes *PTEN* transcription in the mutant nerves. Together, these data argue for the existence of multiple distinct mechanisms of TSC1/2 deficiency for downregulation of AKT activity in SCs (Fig. 4h).

In essence, above negative feedback mechanisms allowed us to study nerve development and SC myelination under the exclusive constellation of mTORC1 hyperactivity and concurrently suppressed AKT signaling. Thus, the characterization of TSC2-SCKO and TSC1-SCKO mice for the first time sheds light on discrete mTORC1 functions in SCs independent from AKT activity. By contrast, nerves from PTEN-SCKO mice display increased AKT activity along with mTORC1 elevations. Moreover, rapamycin administration in TSC2-SCKO mice augments AKT activity reciprocally to the downregulation of mTORC1 signaling owing to the release of inhibitory feedback on AKT [24]. Therefore, phenotypic differences between these models as well as phenotypic variation after rapamycin application may also help to reveal discrete roles of AKT signaling in developing SCs *in vivo*. In fact, a recent study in transgenic mice proposed that AKT exerts mTORC1-independent functions in developing SCs including axon sorting and wrapping by dynamic SC extensions through the activity of Rac1 [11]. Rac1 is a small G protein previously shown to promote the generation of radial SC lamellopodia required for the initiation of myelination [44,45]. This is consistent with our data demonstrating that TSC2-deficient SCs wind around individual axons only incompletely during development (resulting in ‘naked’ axons), which is also associated with a paucity of SC cytoplasmic processes between axonal profiles on ultrastructural level [24]. Additionally, both TSC2-SCKO and TSC1-SCKO nerves show axonal sorting defects with abnormal presence of large diameter axons in bundles of small caliber axons leading to aberrant Remak bundle structure [24]. This suggests the possibility that mutant SCs are incapable of extracting such axons from these bundles for subsequent myelin ensheathment because of deficient expansion of radial SC lamellopodia secondary to abolished AKT and Rac1 activities. This notion is supported by results demonstrating that reactivation of AKT in TSC2-SCKO nerves is associated with remarkable restoration of such abnormal Remak bundle structure [24]. Conversely, mice with constitutively active AKT display excessive segregation and redundant wrapping of axons, particularly of small diameter fibers in Remak bundles, which is not influenced by mTORC1 inhibition [11]. Interestingly, hyperactivation of AKT in the context of mTORC1 disruption in raptor-deficient SCs also results in axonal sorting defects and abnormal Remak bundles [5].

Given these important functions, the dramatic suppression of AKT activity in TSC2-SCKO mice raises the possibility that it may essentially account for the failure to myelinate axons irrespective of the mTORC1 hyperactivity. A recent study provides evidence against this notion [4]. The authors found that the suppressed AKT signaling in TSC1-deficient SCs undergoing development could be restored by co-ablation of PTEN (in TSC1^fl/fl^; PTEN ^fl/fl^; P0^Cre^ mice), and this manipulation clearly exacerbated the hypomyelination phenotype seen in TSC1-SCKO nerves. Notably, the restored AKT signaling was accompanied by further increases in mTORC1 activity in mutant SCs. This underscores that hyperactive mTORC1 is the detrimental factor for myelination.

Strikingly, as opposed to the mTORC1-driven glial overproliferation, we demonstrated a completely distinct phenotypic outcome if mTORC1 hyperactivity is evoked in already differentiated SCs. This unveils a different function of mTORC1 signaling in the SC lineage. Under conditions when TSC2- or TSC1-deficient SCs can mature and initiate myelination, mTORC1 upregulation eventually results in disproportional and ectopic growth of myelin sheaths [24]. On nerve transverse sections such inappropriate growth appears as redundant apposition of myelin in form of myelin outfoldings, recurrent myelin loops, and focal myelin thickening. Again, such focal hypermyelination occurs under settings of markedly repressed AKT activity in the TSC1/2 mutants, which likely accounts for the increased cumulative g-ratios in mutant nerves, a measure reflecting overall thinner myelin sheaths [24]. We therefore propose that adequate assembly of myelin with proper myelin sheath geometry requires AKT and mTORC1 to act in concert. Thus, mTORC1 hyperactivity decoupled from parallel AKT signaling results in aberrant, focal myelin growth and distorted myelin sheaths. In the context of normal nerve development, we suggest the model that mTORC1 promotes the biosynthesis of myelin constituents as such (i.e. myelin lipids and proteins), while AKT together with other pathways is required for coordinating the correct incorporation of these building blocks into newly generated SC membranes to be wound around axons.

In light of these data and considerations we propose the model shown in Fig. 5 for the distinct but cooperative functions of AKT and mTORC1 in the SC lineage. During early nerve development, high mTORC1 activity in SC precursors is primarily required for optimal stimulation of glial proliferation. Alongside mTORC1, high AKT activity promotes glial extraction of individual large diameter axons from immature fiber bundles and then drives spiral enwrapping of such axons by expanding SC projections. At this stage AKT is also important to properly engulf small diameter axons to ensure normal architecture of nascent Remak bundles. A tight cooperation between both signaling components is likely already at work here in view of the requirement of correct SC proliferation for axonal sorting [3]. Subsequently, to prevent exuberant wrapping of axons and to exit the cell cycle for proper differentiation of SCs, AKT and mTORC1 activities are gradually restrained. This decrease allows time to consolidate initial compact myelin sheaths by dampening the continual myelin production. Next, in the manner described above, declining AKT and mTORC1 activities cooperatively regulate further growth of myelin sheaths in order to establish normal myelin sheath geometry. It is clear from this model that separate elevations of mTORC1 signaling can lead to abnormal SC proliferation or aberrant myelin synthesis depending on the time point of induction (i.e. prior or after terminal differentiation of SCs). Although this may prove to be a daunting task, in the future it will be important to systematically test the proposed AKT roles separated from mTORC1 signaling changes *in vivo*, for example by characterization of mouse mutants with hyperactive or inactive AKT decoupled from mTORC1 activity changes in developing and mature SCs.

**Figure 5.**
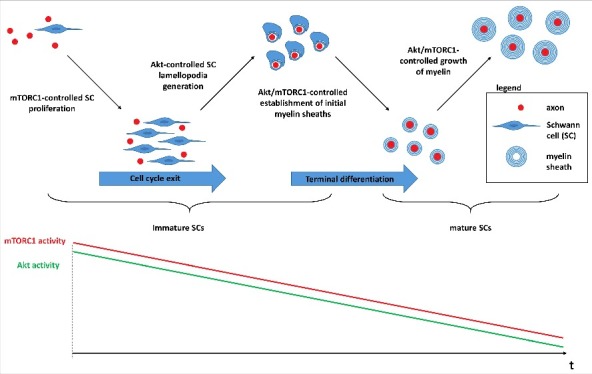
Model for the cooperative functions of AKT and mTORC1 in myelinating SCs. During early nerve development mTORC1 is a critical regulator for the optimal proliferation of SC precursors through control of cycling proteins and the CDK inhibitor p27^Kip1^. The concurrent high AKT activity promotes axon sorting and adequate wrapping via Rac1-dependent mechanisms that drive the formation of SC lamellopodia. The progressive downregulation of mTORC1 activity (bottom schematic graphs) is then necessary for subsequent cell cycle exit and the transition of SC precursors and promyelinating SCs to mature SCs that elaborate compact myelin sheaths around axons. In parallel, the high AKT activity is progressively downregulated to prevent inappropriate sorting and wrapping of axons. The fine tuning of residual activities of AKT and mTORC1 is important to eventually establish normal myelin sheath geometry, as the thickness of the myelin sheath is adjusted to axonal size in order to ensure optimal nerve conduction. Deviations from this temporal pattern of AKT and mTORC1 activities in SCs can have distinct phenotypic outcomes for myelination. Most prominently, induction of glial mTORC1 hyperactivity in SC precursors causes aberrant proliferation prohibitive for myelination, whereas mTORC1 induction after the transition to mature myelinating SCs results in abnormal growth of the myelin sheath.

Taken together, the work we lay out here provides important insights into the central role of PI3K/AKT-mTORC1 signaling axis in nerve development and myelination. It is likely we are just scratching the surface with regard to the regulations and functions of this intriguing signaling network. A particularly looming question is which upstream factors in SCs including bioenergetic and nutritive components regulate the activity of mTORC1. We anticipate that elucidating the molecular underpinnings for regulation of AKT and mTORC1 activities in SCs together with their crosstalk will foster the development of novel therapeutics to treat various peripheral neuropathies. This field will certainly benefit from the increasingly sophisticated understanding of how mTORC1 functions, and recent seminal discoveries surrounding the roles of mTORC1 in aging, cancer biology, and neurodegeneration.

## Materials and methods

### Generation of mutant mice lacking TSC2 in SCs

All animal experiments were reviewed and approved by the Roswell Park Cancer Institute (RPCI) Institutional Animal Care and Use Committee (protocol approval UB1301M). To generate mice with perinatal ablation of TSC2 exclusively in SCs, floxed TSC2 mice [46]. were crossed to P0-Cre transgenic mice [25]. Littermates carrying floxed alleles, but lacking Cre expression, were used as controls. Genotyping was performed by PCR strategies using standard procedures and appropriate primers (sequences available upon request).

### Organotypic DRG neuron/SC co-cultures

Mouse DRG neurons were isolated from E13.5 embryos, trypsinized, mechanically dissociated, and plated on matrigel-coated 12mm glass coverslips in C-media (DMEM, 10% FBS, 4 mg/ml glucose, 2 mM L-glutamine, 50 ng/ml NGF, and P/S). On the next day cultures were switched to neurobasal medium supplemented with B27 and NGF in addition to 4mg/ml glucose and 2mM L-glutamine, with media change every other day. At DIV 7 myelination was induced by adding C-media supplemented with 50 µg/ml ascorbid acid (Sigma Aldrich). At DIV18 co-cultures were briefly fixed with 4%PFA/PBS, and then immunostained with primary antibodies TUJ1 (Covance #PRB-435P), MBP (generated from a rat hybridoma cell line, kind gift from Virginia Lee, University of Pennsylvania), and Alexa 488- and Alexa 568-coupled secondary antibodies. The preparations were counterstained with DAPI and mounted in Vectashield for subsequent microscopic imaging. Analogous staining with primary antibody TSC2 (Cell Signaling Technologies, #4308) instead of MBP was performed in separate experiments prior to the induction of myelination with C-media.

### Nerve histology

Semithin light microscopy and electron microscopy and acquisition of micrographs was performed as described previously [24]. Measurements of sciatic nerve section cross areas were carried out using ImageJ software.

### Quantification of DAPI^+^ nuclei and immunofluorescence

DAPI^+^ nuclei were counted on entire sciatic nerve transverse frozen sections (at least 3 mice per genotype and developmental time-point). Immunofluorescence staining on longitudinal frozen sciatic nerve sections using PTEN primary antibodies (Cell Signaling Technologies #9188) and Alexa 488-conjugated secondary antibodies was performed as described previously [24].

### Western blotting

Western blotting analysis was performed using standard procedures as described previously [24]. The following primary antibodies were used: IRS-1 (Santa Cruz Biotechnology, sc-8038), Grb10 (Santa Cruz Biotechnology, sc-74509), p-Rictor (Thr1335) (Cell Signaling Technologies, #3806), PTEN (Cell Signaling Technologies, #9188), Hif1α (Novus Biologicals #NB100-479), β-actin (Sigma Aldrich, #A2228), GAPDH (Millipore, #MAB374). HRP-coupled secondary antibodies from Cell Signaling Technologies and Jackson Immuno Research were used for signal detection.

### Statistical analysis and presentation of data

Quantitative data are presented as mean ± SEM. Statistical analyses were performed using Graph Pad Prism software. A two-tailed Student's t test was used for group comparisons and statistical significance was considered if P < 0.05.
